# The effects and mechanisms of *SLC34A2* in tumorigenesis and progression of human non-small cell lung cancer

**DOI:** 10.1186/s12929-015-0158-7

**Published:** 2015-07-09

**Authors:** Yu Wang, Weihan Yang, Qiang Pu, Yan Yang, Sujuan Ye, Qingping Ma, Jiang Ren, Zhixing Cao, Guoxing Zhong, Xuechao Zhang, Lunxu Liu, Wen Zhu

**Affiliations:** State Key Laboratory of Biotherapy and Cancer Center, West China Hospital, Sichuan University, and Collaborative Innovation Center for Biotherapy, NO. 1, Keyuan 4th Road, Gaopeng Street, High Technological Development Zone, 610041 Chengdu, Sichuan P. R. China; Department of Thoracic Surgery, West China Hospital, Sichuan University, No. 37 Guo Xue Xiang, 610041 Chengdu, Sichuan P. R. China

**Keywords:** *SLC34A2*, ATII cells, NSCLC, Tumorigenesis and progression, Mechanism

## Abstract

**Background:**

*SLC34A2* with highest expressions in lung, small intestine and kidney encoded a type 2b sodium-dependent phosphate transporter (NaPi-IIb). In lung, *SLC34A2* only expressed in the apical membrane of type II alveolar epithelium cells (ATII cells) and played a pivotal role during the fetal lung development and embryonic development. ATII cells acting as multifunctional stem cells might transform into NSCLC after undergoing exogenous or endogenous factors. Increasing evidences showed that the genes performing critical roles during embryogenesis were also expressed during the development of cancer. In addition, recent research found the expression of *SLC34A*2 had a significant difference between the surgical samples of NSCLC and normal tissues, and *SLC34A2* was down-regulated in lung adenocarcinoma cell line A549 and up-regulation expression of *SLC34A2* could significantly inhibit cell viability and invasion of A549 *in vitro*. These results suggested *SLC34A2* might play an important role in the development of NSCLC. However, the role of *SLC34A2* in tumorigenesis and progression of NSCLC remains unknown.

**Results:**

Our study found that *SLC34A2* was also significantly down-regulated in 14/15 of examined NSCLC tissues. Moreover, we found that expressions of *SLC34A2* were reduced in six NSCLC cell lines for the first time. Our result also revealed a dramatic inhibitory effects of *SLC34A2* on cell growth, migration and invasion of several NSCLC cell lines. *SLC34A2* also strongly inhibited tumor growth and metastasis ability in A549 subcutaneous tumor model and lung metastasis model, respectively. Further studies found that the suppressive effects of *SLC34A2* on tumorigenesis and progression might be associated with the down-regulation of related protein in PI3K/Akt and Ras/Raf/MEK signal pathway.

**Conclusions:**

For the first time, our data indicated that *SLC34A2* could exert significantly suppressive effects on tumorigenesis and progression of NSCLC. *SLC34A2* might provide new insights for further understanding the early pathogenesis of human NSCLC.

## Background

Despite advances in early detection and standard treatment, NSCLC still has a poor 5-year survival and it is often diagnosed at an advanced stage [1]. Therefore, there is a great need to understand the pathogenesis of NSCLC clearly in order to define new biomarkers of early diagnosis and find new targets for NSCLC therapy.

Human *SLC34A2* cDNA was first isolated and cloned from a human small intestine and lung cDNA library respectively in 1999 [2, 3]. *SLC34A2* encodes a type 2b sodium-dependent phosphate transporter, NaPi-IIb. It is a multi-pass membrane protein, composed of 690 amino acids. This protein has been reported to mediate transporting inorganic phosphate into epithelial cells via sodium ion co-transport and have a role in the synthesis of surfactants in lung alveoli [4]. Recent studies pointed that although *SLC34A2* was expressed in various human tissues, the highest expressions were shown in lung, small intestine and kidney [3, 5]. In lung, expression of *SLC34A2* was only found in the apical membrane of type II alveolar epithelium cells (ATII), thus it could be regarded as a candidate specific marker for ATII cells [4–6]. *SLC34A2* played a significant role in ATII cells [6]. The anomalous expression of *SLC34A2* might result in functional disorder of ATII cells. Some research showed that mutations in *SLC34A2* caused Pulmonary Alveolar Microlithiasis (PAM) [7] and anomalous expression of *SLC34A2* was responsible for some other diseases such as hypophosphatemia, infertility and Testicular Microlithiasis (TM) [7, 8]. Besides, recent research reported that *SLC34A2* was down-regulated in breast cancer, but overexpression of *SLC34A2* was detected in ovarian cancer and papillary thyroid cancer [8]. These studies indicated that *SLC34A2* was related to tumorigenesis and progression. However, the researches about the function of *SLC34A2* in tumorigenesis and development, especially the relationship between *SLC34A2* and lung cancer, have not been reported until now.

Recently, Eugene P. Kopantzev reported the expression of *SLC34A2* in human lung development. The expression of *SLC34A2* was augmented in human fetal lung development, and reached highest level at the canalicular stage of lung development which remained unchanged during further development [9]. Meanwhile, Mitsuyoshi Hashimoto observed that *SLC34A2* was first faintly detected on gestational day 16.5, but rapidly augmented after gestational day 18.5 in the developing rat lung, finally kept the constant level even after postnatal day until adult [5]. Moreover, *SLC34A2* was essential for embryonic development. Homozygous *SLC34A2* deficient mice died in uterus soon after implantation. NaPi-IIb was detected at the point where embryonic and maternal circulations were in closest contact [10]. These results suggested that *SLC34A2* locating in AT-II cells played a pivotal role during the fetal lung development and embryonic development.

Increasing evidents showed that genes performing critical roles during embryogenesis were also expressed during the development of cancer, especially genes which were associated with deprogramming and maintaining the undifferentiated stem cell state [11, 12]. For example, *Ovca1* is a tumor suppressor that can modify p53-induced tumorigenesis and participate in the tumorigenesis. Moreover, *Ovca1–2* or *Ovca1* homozygous mutants died soon after birth, which suggested that *Ovca1* was required for embryonic development and postnatal viability [13]. Therefore, we supposed that *SLC34A2*, which played a pivotal role in embryonic development and the fetal lung development, might participate in the tumorigenesis and progression of lung cancer.

Otherwise, recent researches showed that ATII cells could serve as the stem cells of the alveolar epithelium [14]. Moreover, Gurmukh [15] showed that lung carcinomas exhibited the feature of ATII cells, like the presence of lamellar bodies in the cytoplasm and tubular electron-microscopic nuclear inclusions. Meanwhile, they identified the existent of ATII cells in those lung carcinomas by immunoperoxidase staining with surfactant specific apoprotein. In addition, Kitinya [16] and Gazdar [17] found that large cell carcinoma and adenocarcinoma histogenetically were related to ATII cells by using monospecific IgG against pulmonary surfactant apoprotein. These evidences demonstrated that ATII cells might be the cell of origin of several types of lung cancer including large cell carcinoma and adenocarcinoma [16, 17]. Because in lung, the expression of *SLC34A2* was only found in ATII cells and ATII cells might be the origin of several types of lung cancer. These facts further suggested that *SLC34A2* might play an important role in tumorigenesis of NSCLC.

Particularly in 2008, Eugene P. Kopantzev demonstrated that the expression of *SLC34A2* in human normal lung tissue was ten times higher than that in surgical samples of NSCLC [9]. Foremost, our recent research found that expression of *SLC34A2* was down-regulated in lung adenocarcinoma cell line A549 and elevated expression of *SLC34A2* could significantly inhibit cell viability and invasion of A549 *in vitro* [18]. However, until now, there are not any reports about the effects of *SLC34A2* on tumorigenesis and development of NSCLC.

To explore the effects of *SLC34A2* on tumorigenesis and development of NSCLC, the expressions of *SLC34A2* in 15 NSCLC tissues and 6 NSCLC cell lines were examined in this study firstly. Secondly, effects of *SLC34A2* on NSCLC cell proliferation, apoptosis, migration and invasion were investigated *in vitro*. Thirdly, effects of *SLC34A2* on NSCLC growth and metastasis ability were further identified with A549 subcutaneous xenotransplanted tumor model and lung metastasis model. Finally, potential mechanisms of *SLC34A2* were further evaluated. For the first time, our study focused on the effects of *SLC34A2* on tumorigenesis and development of NSCLC. It will be helpful for deeply understanding the tumorigenesis and pathogenesis of NSCLC.

## Methods

### Cell lines and animals

The NSCLC cell lines A549, H1299, H460 and Human bronchial epithelial cell HBE were purchased from American Type Culture Collection (ATCC, Manassas, VA, USA). The NSCLC cell lines H358, 95D and SK-MES-1 were purchased from Cell Bank of the Chinese Academy of Sciences (Shanghai, China). The cell lines A549, H1299, H460 and H358 were maintained in RPMI-1640 (Gibco Laboratories, Grand Islands, NY, USA) containing 10 % heat-inactivated fetal bovine serum (FBS) (Gibco, Gaithersburg, USA). The cell lines HBE, 95D and SK-MES-1 was maintained in DMEM (Gibco Laboratories, Grand Islands, NY, USA) supplemented with 10 % heat-inactivated FBS (Gibco, Gaithersburg, USA). All cells were cultured at 37 °C in a humidified atmosphere of 5 % CO_2_. Female athymic BALB/c *nu/nu* mice (HFK Bioscience, Beijing, China), 3–4 weeks old were maintained at the Animal Core Facility at West China Hospital, Sichuan University under specific pathogen-free (SPF) condition. All studies on mice were conducted in accordance with the National Institutes of Health Guide for the Care and Use of Laboratory Animals.

### Tissue specimens

NSCLC tumor and normal adjacent NT tissue specimens were obtained from 15 patients from Department of Thoracic Surgery, West China Hospital, Sichuan University in 2011. This study was performed with the approval of the Medical Ethical Committee of West China Hospital, Sichuan University. Ages of the patients ranged from 50 to 81 years (median: 64 years), with a male-to-female ratio 1:2. The tumor specimens encompassed 12 adenocarcinoma and 3 squamous cell carcinoma. Tumor specimens were staged according to TNM classification (2007): stage I ~ II (10 cases), III (4 cases) and IV (1 case). Tumor samples were clinically characterized (Table 1).

**Table 1:** Patient clinical features and SLC34A2 expression profile

Patient No.	Age	Gender	Histological grade	Clinical stage	Normalised *SLC34A2* amount in tumour tissue relative to adjacent normal tissue 2^-ΔΔCt^	Lymph node metastatic or non-metastatic
1	54	F	A	IIIA	0.5675	Yes
2	53	F	A	IA	0.0715	No
3	74	F	A	IIA	0.0585	Yes
4	71	M	A	IB	0.1675	No
5	52	F	A	IB	0.2573	No
6	59	F	A	IIIB	0.2829	Yes
7	79	F	A	IB	0.4157	No
8	74	F	A	IB	0.3364	No
9	61	F	A	IIIA	0.1702	Yes
10	69	F	A	IIB	0.4994	No
11	64	F	A	IIA	3.3600	No
12	67	M	A	IIA	0.6622	No
13	81	M	S	IV	0.2813	Yes
14	50	M	S	IIA	0.1462	No
15	55	M	S	IIIA	0.5016	Yes

a. *M* male, *F* female, *A* adenocarcinoma, *S* squamous cell carcinoma. b. Relative quantification was performed by the 2^-ΔΔCt^ method with the adjacent normal lung tissue sample as a calibrator. Data show the means from three independent analyses. Every independent analysis was carried out after the RNA extraction step. Total RNA was poly-A tailed, reverse transcripted, and then real-time PCR tested. ΔC_T_ obtained from real-time PCR was subject to paired *t*-test (ΔC_T_ = C_*TSLC34A2*_-C_Tβ-actin_). The expression levels of SLC34A2 in tumor tissues were significantly lower than adjacent normal tissues (*P* < 0.05)

### Quantitative real-time PCR (qPCR) analysis

Total RNA from tissues and cultured cells was isolated by using Trizol reagent (Invitrogen, Carlsbad, CA, USA) according to the manufacturer’s protocol. Synthesis of cDNA with reverse transcriptase was performed by PrimeScript RT reagent Kit Perfect Real Time (TaKaRa, Dalian, China). For *SLC34A2* gene expression analysis, quantitative real-time PCR analysis was done in the iCycler iQ5 real-time PCR Detection system (Bio-Rad Laboratories, Hercules, CA, USA) with SYBR Green Reagents (Bio-Rad, CA). Cycling conditions were used according to the manufacturer’s instructions: 95 °C for 3 min followed by 50 cycles of 95 °C for 10s 58 °C for 20s and 72 °C for 2 min. Comparative gene expression analysis was performed using the 2^-ΔΔCt^ method with normalization to the level of internal control gene *GAPDH*. The primer sequences used are as follows: *SLC34A2* (F): 5’-GAGAACATCGCCAAATGC-3’ (R): 5’-GCAACCACAGAGGACCAG-3’.

### Construction and preparation of plasmids

According to the *SLC34A2* cDNA coding sequence (GeneBank, NM**_**006424), the upstream primer *SLCS4A2* (F: 5’- GCGGATCCTAATGGCTCCCTGGCCTGAAT-3’) and downstream primer *SLCS4A2* (R: 5’-GCGAATTCCTACAAGGCCGTGCATTCG -3’) were used to clone CDS sequence of *SLCS4A2* from the original cloning vector, pcmv-sport6-*SLCS4A2* (Open Biosystems, USA). The cloned cDNA sequence was connected to the eukaryotic expression vector pcDNA3.1 (+) (Invitrogen, Carlsbad, CA, USA) by DNA Ligation Kit Ver.2.0 (TaKaRa, Dalian, China) to form a reconstructed plasmid named pcDNA3.1-*SLC34A2*. Pure pcDNA3.1 and pcDNA3.1-*SLC34A2* plasmids were prepared using Tenderfe^et^ Plasmid Giga Kit (Siegen, Chatsworth, CA, USA) for the following experiments. The construct was confirmed by DNA sequence analysis to verify its identity and absence of mutation.

### Transient transfection

The NSCLC cell lines A549, H1299, H358, H460, 95D and SK-MES-1 were transfected with either pcDNA3.1-*SLC34A2* recombinant or pcDNA3.1 vector-alone plasmids with Lipofectamine2000 Reagent (Invitrogen, Carlsbad, CA, USA) according to manufacturer’s instruction. Cells transfected with pcDNA3.1-*SLC34A2* or pcDNA3.1 were named as P-S or P respectively. Untransfected cells were named as Control.

### Stable transfection

For stable transfection of *SLC34A2* in A549 cells, A549 cells were prepared in 6-well plates. When cell density reached 60–70 %, they were transfected with the plasmid pcDNA3.1-*SLC34A2* or pcDNA3.1 using Lipofectamine2000 (Invitrogen, Carlsbad, CA, USA). The medium was replaced after 48 h and G418 (800 μg/ml) (Sigma, CA, USA) was added to select stable transfectants. After 14 days, G418 sulfate (geneticin)-resistant colonies were isolated by medium containing G418 and expanded. The *SLC34A2*-positive transfected cells were identified by western blot. Stable transfectants were maintained in the medium containing G418 (500 μg/ml). A549 cells with stably expressing pcDNA3.1-*SLC34A2* and pcDNA3.1 were named as A549-pcDNA3.1-*SLC34A2* (A549-P-S) and A549-pcDNA3.1 (A549-P) respectively for further analysis.

### Measurement of inorganic phosphate

After transient transfection with pcDNA3.1-*SLC34A2*, the phosphorous concentration in supernatant of medium was measured using a phosphorous measurement kit (Jiancheng Bioengineering Institute, Nanjing, China) according to the manufacturer’s instructions. RIPM-1640 or DMEM medium was taken as a comparison.

### Cell viability assay

To investigate the effects of *SLC34A2* on proliferation of NSCLC cells *in vitro*, A549, H1299, H460, H358, 95D and SK-MES-1 cells were transfected with pcDNA3.1-*SLC34A* or pcDNA3.1 respectively. Cells untransfected served as controls. After 48 h treatments, cell medium was replaced with 20 ml of MTT (3-(4,5-dimethylthiazol-2-yl)-2,5-diphenyltetrazolium bromide; Sigma, St Louis, MO, USA) in each well, together with 180 ml RPMI1640, and incubated for another 4 h. The cell viability was evaluated by MTT assay. The percentage of viable cells was calculated in terms of the absorbency of treated cells relative to that of untreated cells.

### Cell apoptosis ratio assay

After transient transfection for 48 h, the cell apoptosis ratio was determined with Annexin-V and PI staining Kit (KeyGEN, Nanjing, China) by Fluorescence-activated cell sorting (FACS) according to the manufacturer’s instructions. Untransfected cells served as controls.

### *In vitro* migration and invasion assays

After transient transfection, migration and invasion assays were performed using millicell chambers of 8 μm pore size (Millipore, Billerica, MA) and Boyden Chambers (BD Biosciences, USA) with matrigel coated before using, respectively. Briefly, for migration, transfected cells were resuspended in serum-free medium, and 200 μl of the cell suspension (5 × 10^4^ cells) was added to the upper chamber, the lower compartments were filled with medium containing 1 % FBS. The chamber was then cultivated in 5 % CO_2_ at 37 °C for 24 h. For invasion assays, 2 × 10^5^ cells in 1 % FBS medium were added to the upper chamber, and the lower compartments were filled with medium containing 10 % FBS respectively. After incubating for 24 h, cells that had attached to the lower surface of the filter were fixed and stained with 0.1 % crystal violet. Cells untransfected served as controls. The numbers of the migrating and invading cells were calculated by counting the mean number of six random areas under a light microscope at 200× magnification.

### *In vivo* tumorigenicity assay

All groups of cells were injected subcutaneously into the right flank of nude mice with the concentration of 7.5 × 10^6^ cells/0.1 ml per mouse. Each group contained 3 mice. Tumor size was measured every 2d by using a venire calliper. The tumor volume was calculated by the formula 1/6πab^2^ (π = 3.14; a = long axis and b = short axis of the tumor). About sixty days after injection, animal were sacrificed and flank tumors were removed and weighed. Half of each group of tumor samples were fixed in 4 % paraformaldehyde for histological analysis, and the others were harvested immediately and freshly frozen.

### Lung metastasis model

All groups of cells were injected intravenously via tail vein with the concentration of 3 × 10^6^ cells/0.2 ml per mouse. Each group contains 6 mice. Four weeks later, lungs from the mice of the three groups were injected intratrachealy with India ink and fixed in AAF solution, and the number of surface lung metastatic nodules was counted under a dissecting microscope.

### Western blot analysis

Cell lysates from A549, 95D, A549-P-S, A549-P, 95D-P-S, 95D-P cells were prepared by extracting proteins with RIPA buffer (Pierce) containing protease inhibitor cocktail set I (Merck KGaA, Germany). The protein concentrations were measured by the BCA protein assay reagent (Pierce Biotechnology, Rockford, IL, USA). An equal amount of total proteins was loaded on each lane and separated by SDS-PAGE and subsequently blotted on a PVDF membrane (pore size, 0.45 am; Millipore, Billerica, MA). Membranes were incubated with blocking buffer for 60 min at room temperature and then incubated with a specific primary antibody against SLC34A2 (Sent Cruz Biotechnology, Santa Cruz, CA, USA), Cycling D3, PI3 Kinas, phospho-Akt (Ser473), Akt, phospho-mTOR (Ser2448), mTOR, phospho-MEK1/2 (Ser217/221), MERK1/2, phospho-p44/p42 MAPK (Erk1/2) (Thr202/Tyr204), Erk1/2 (Cell Signal pathway Technology, Beverly, MA, USA) and GAPDH (Sigma, CA, USA) with Blotto overnight at 4 °C. The membranes were washed and incubated with a horseradish peroxidase (HRP)-conjugated secondary antibody. Blots were then exposed to secondary antibodies and visualized by an enhanced chemiluminescence (ECL) blotting analysis system (GE Healthcare Life Sciences, Piscataway, NJ).

### Statistical analysis

All results represented the arithmetic mean ± SD standard error of triplicate determinations of at least three independent experiments done under the same conditions. The significant difference of the experimental results was calculated using one-way ANOVA and an unpaired Student’s *t*-test with SPSS13.0 software. *P* < 0.05 was considered as statistically significant.

## Results

### Expression of *SLC34A2* in human NSCLC tissues and cell lines

**Fig. 1:**
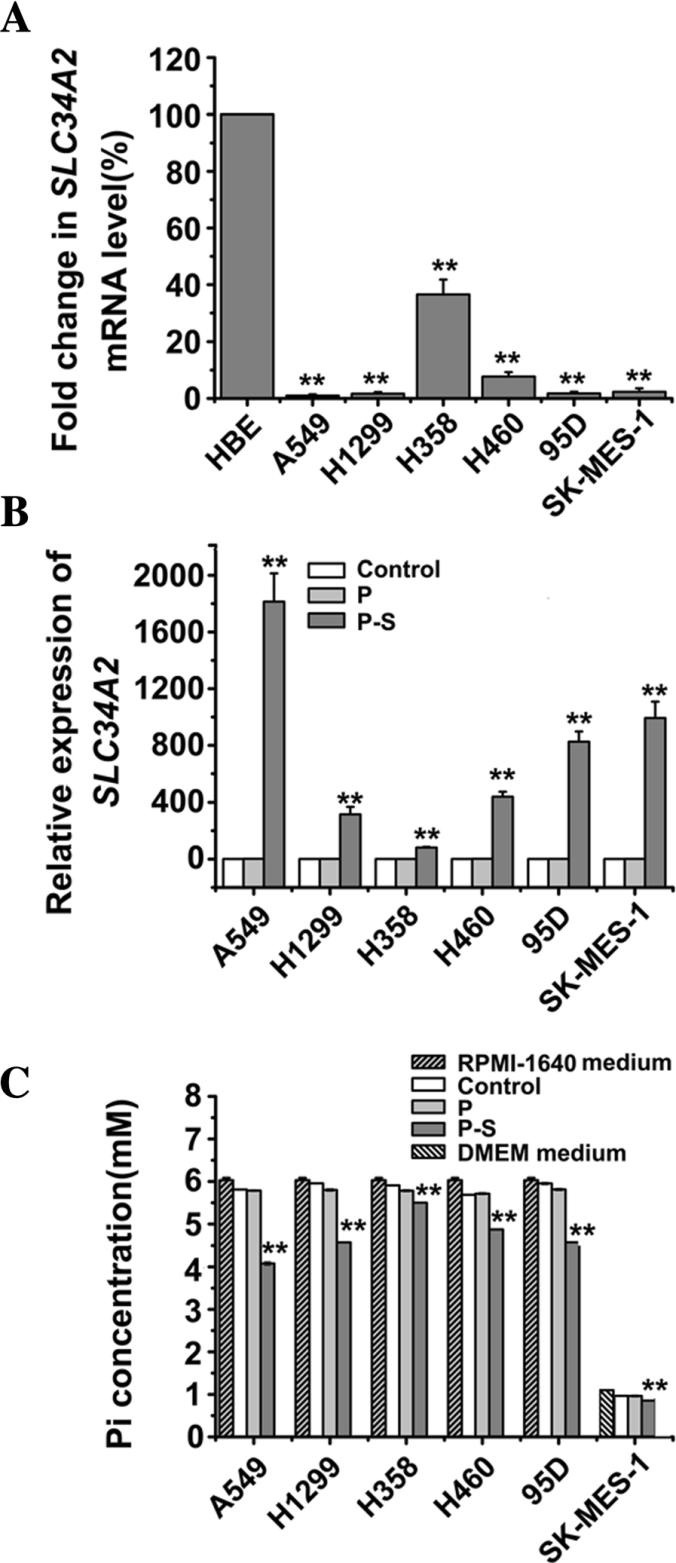
*SLC34A2* expression was down-regulated in NSCLC cell lines and *SLC34A2* was re-expressed in *SLC34A2*-transfected cells. **a** mRNA levels of *SLC34A2* in six NSCLC cell lines were determined by RT-PCR. HBE cells served as control. **b** mRNA levels of *SLC34A2* in six NSCLC cell lines were detected by RT-PCR after transfected with pcDNA3.1-*SLC34A2* (P-S) or pcDNA3.1 (P) for 48 h, untransfected cells was named as Control. **c** The concentration of phosphorous in the supernatant of medium was measured by using a phosphorous measurement kit after 48 h transient transfection

To determine expression levels of *SLC34A2* in human NSCLC, RT-PCR was done in 15 matched NSCLC tumor/ corresponding adjacent normal tissues and 6 NSCLC cell lines, including A549, H1299, H358, H460, 95D and SK-MES-1. As shown in Table 1, the mRNA expression levels of *SLC34A2* were notably down-regulated in NSCLC tumor tissues compared with their normal counterparts. The expression levels of *SLC34A2* showed no difference among age, gender, case type, TNM staging or metastasis in 15 tested lung cancer tissues. In addition, our result also found that the expressions of *SLC34A2* in A549, H1299, H358, H460, 95D and SK-MES-1 cells were reduced by 100.52, 59.02, 1.74, 11.95, 55.73, 42.07 fold, respectively, compared to HBE (Fig. 1a). These data showed that the expressions of *SLC34A2* were frequently down-regulated in NSCLC tissues as well as in NSCLC cell lines. It indicated that the down-regulation of *SLC34A2* might be involved in the tumorigenesis of NSCLC.

### Expression and Function after transient transfection of *SLC34A2* cDNA

Firstly, the expression levels of *SLC34A2* in A549, H1299, H358, H460, 95D and SK-MES-1 cell lines with pcDNA3.1*-SLC34A2* transient transfection for 48 h were determined by quantitative real-time PCR. As shown in Fig. 1b, *SLC34A2*-transfected cells (P-S) expressed significant high mRNA levels of *SLC34A2* compared with untransfected cells (Control) and pcDNA3.1-transfected cells (P) (*P* < 0. 01). And the expressions of *SLC34A2* in 6 *SLC34A2*-transfected cells (P-S):A549, H1299, H358, H460, 95D and SK-MES-1 cells were significantly increased by 1813.43, 313.86, 80.28, 438.96, 824.88, 991.84 fold respectively, compared with matched untransfected cells (Control). These results indicated the exogenous *SLC34A2* could be efficiently expressed in 6 NSCLC cell lines after transient transfection.

To verify whether the *SLC34A2* was functional available in *SLC34A2*-transfected cells, Phosphorous concentration in the supernatant of medium was measured using a phosphorous measurement kit after 48 h transient transfection. The phosphorous concentration in the supernatant was reduced in *SLC34A2*-transfected cells compared with untransfected cells (Control) and vector-transfected cells (P) (*P* < 0. 01) (Fig. 1c). The phosphorous concentration in the supernatant of *SLC34A2*-transfected cells (P-S): A549, H1299, H358, H460, 95D and SK-MES-1 cells were reduced by 0.30, 0.24, 0.07, 0.14, 0.23, 0.12 fold, respectively, compared with matched untransfected cells (Control) and vector-transfected cells (P). These results further showed that *SLC34A2* could be efficiently expressed and normally function its transportion of phosphate after transient transfection in 6 NSCLC cell lines.

### Effect of *SLC34A2* on proliferation and apoptosis *in vitro*

**Fig. 2:**
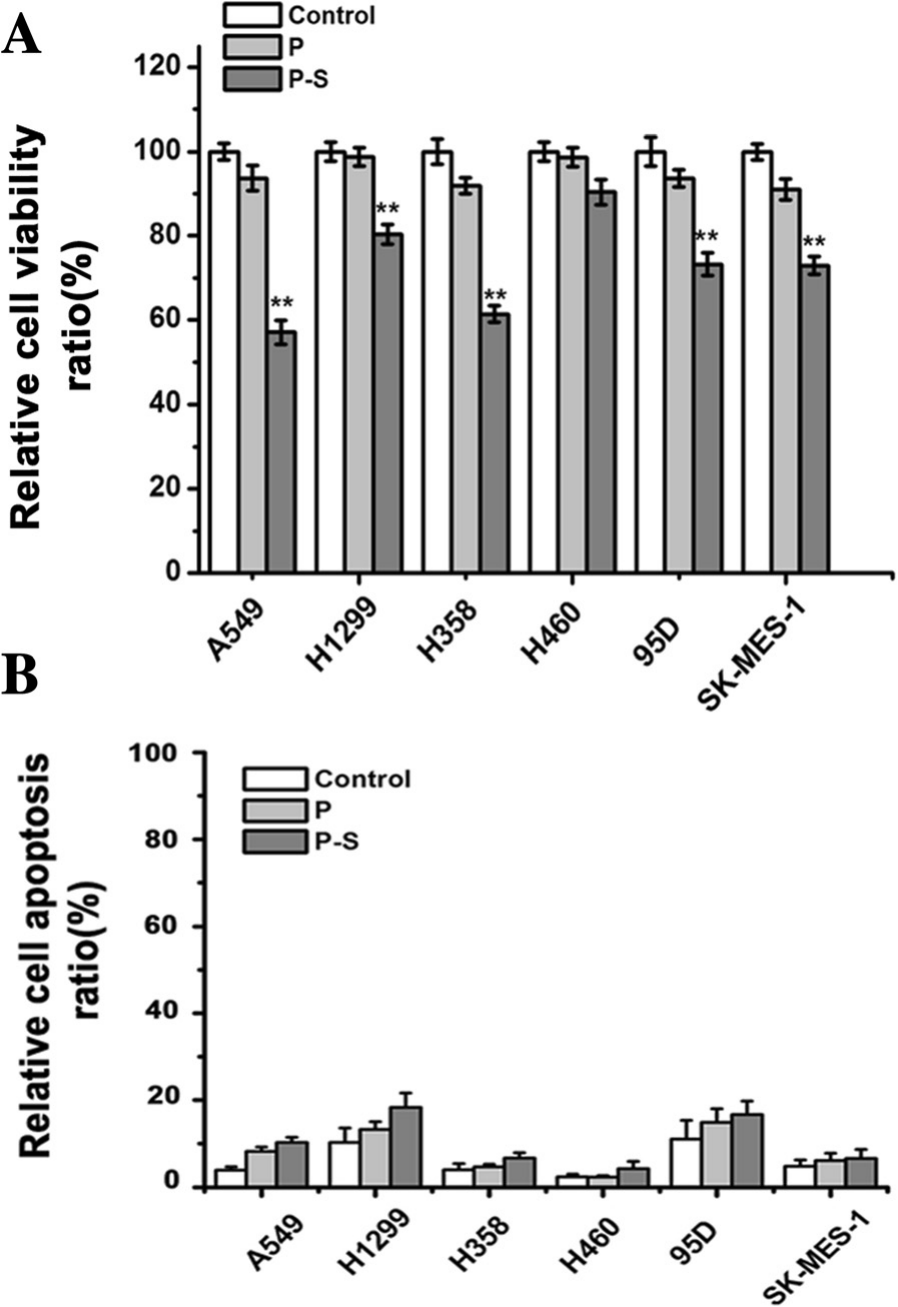
*SLC34A2* inhibited cell growth but had no effect on apoptosis. **a** Cell viability assay. A549, H1299, H460, H358, 95D and SK-MES-1 cells were transfected with pcDNA3.1-*SLC34A2* or pcDNA3.1 respectively. After 48 h treatments, living cells were detected by MTT assay. **b** After transient transfection for 48 h, cell apoptosis ratio was determined with Annexin-V and PI staining Kit by Fluorescence-activated cell sorting (FACS)

To investigate the effects of *SLC34A2* on proliferation of NSCLC cells *in vitro*, A549, H1299, H460, H358, 95D and SK-MES-1 cells were transfected with pcDNA3.1-*SLC34A2* respectively. After 48 h treatment, living cells were detected by MTT assay. As shown in Fig. 2a, compared with untransfected (Control) and pcDNA3.1-transfected cells (P), the significant inhibition of cell proliferation after treated with pcDNA3.1-*SLC34A2* was observed in A549, H1299, H358, 95D and SK-MES-1 cell lines. The growth ratio of A549, H1299, H358, 95D and SK-MES-1 cell lines was significantly reduced by 42.9 %, 19.6 %, 38.6 %, 26.7 %, 27.1 %, respectively, compared with untransfected cells. However, MTT assay revealed that SLC34A2 had no effect on proliferation of H460 cells. These results indicated that except for H460, exogenous expression of SLC34A2 significantly inhibited the viability of A549, H1299, H358, 95D and SK-MES-1 cells *in vitro.*

To determine the effect of *SLC34A2* on apoptosis of NSCLC *in vitro*, we examined Annexin V positivity by fluorescence-activated cell sorting (FACS). As shown in Fig. 2b, the ratio of apoptosis in A549, H1299, H358, H460, 95D and SK-MES-1 cell lines transfected with *SLC34A2* was just increased by 6.40 %, 8.10 %, 2.70 %, 1.87 %, 5.67 % and 1.80 % respectively, compared with untransfected cells. As a result, although increasing expression of *SLC34A2* significantly inhibited the viability of A549, H1299, H358, 95D and SK-MES-1 cells *in vitro*, the effects of *SLC34A2* on apoptosis of these NSCLC cell lines were not obvious.

### Effect of *SLC34A2* on migration and invasion *in vitro*

**Fig. 3:**
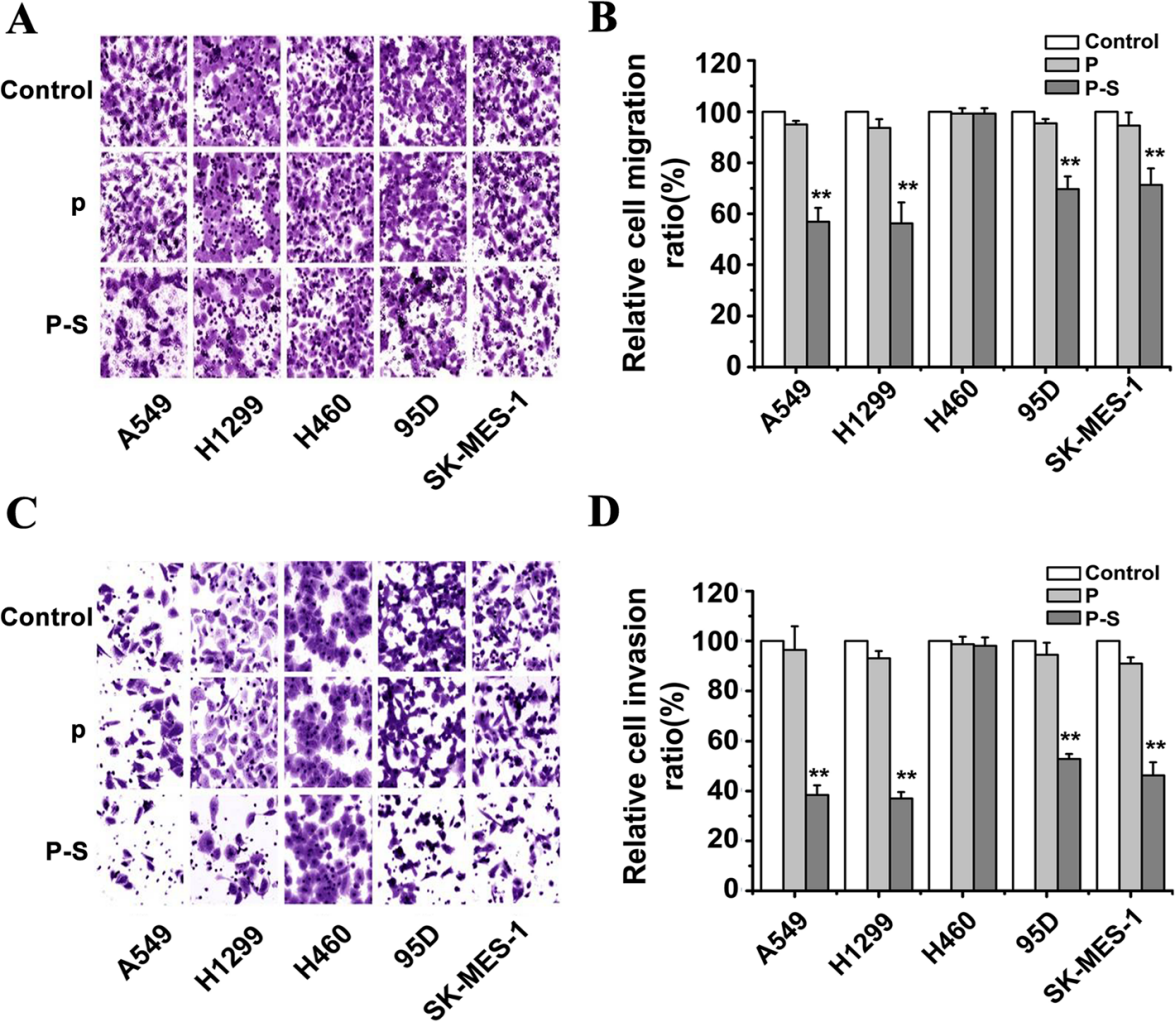
*SLC34A2* inhibited migratory and invasive potential after transient transfection for 48 h. **a** Millicell chamber assay showed reduced cell migratory ability in *SLC34A2*-transfected A549, H1299, 95D and SK-MES-1 cells (P-S) compared with untransfected (Control) and vector-transfected cells (P). **b** Cell migration ratio of these five NSCLC cell lines was assessed by Matrigel invasion assay. **c** Matrigel invasion assay showed depressed cell invasive ability in *SLC34A2*-transfected A549, H1299, 95D and SK-MES-1 cells compared with untransfected and vector-transfected cells. **d** Cell invasion ratio of these five NSCLC cell lines was assessed by Millicell chamber assay

To explore the role of *SLC34A2* on migration and invasion of NSCLC cell lines, A549, H1299, H460, 95D and SK-MES-1 were transiently transfected with pcDNA3.1-*SLC34A2* or blank vector *in vitro*. Then millicell chamber assay and matrigel invasion assay were performed to determine the invasiveness and migration capability. As shown in Fig. 3a, *SLC34A2*-transfected cells (P-S) had significantly less motile compared with untransfected (Control) and blank vector-transfected cells (P). The ratio of cell migration in A549, H1299, 95D and SK-MES-1 cells transfected with *SLC34A2* was decreased by 43.16 %, 43.81 %, 30.33 %, and 28.68 % respectively, compared with the blank control group (Fig. 3b). In addition, as shown in Fig. 3c, the ability of invasiveness in *SLC34A2*-transfected cells was considerably lower than that in negative control group. The invasion capability of *SLC34A2* -transfected cells was suppressed by 61.59 %, 63.01 %, 47.15 % and 53.75 % respectively (Fig. 3d). However, no significant effect of *SLC34A2* on migratory and invasive ability was found in H460 cells. These data showed that except for H460, *SLC34A2* played an important role on reduction of migration and invasion potential in these NSCLC cells *in vitro.*

### Expression and function after stable transfection of *SLC34A2* cDNA

**Fig. 4:**
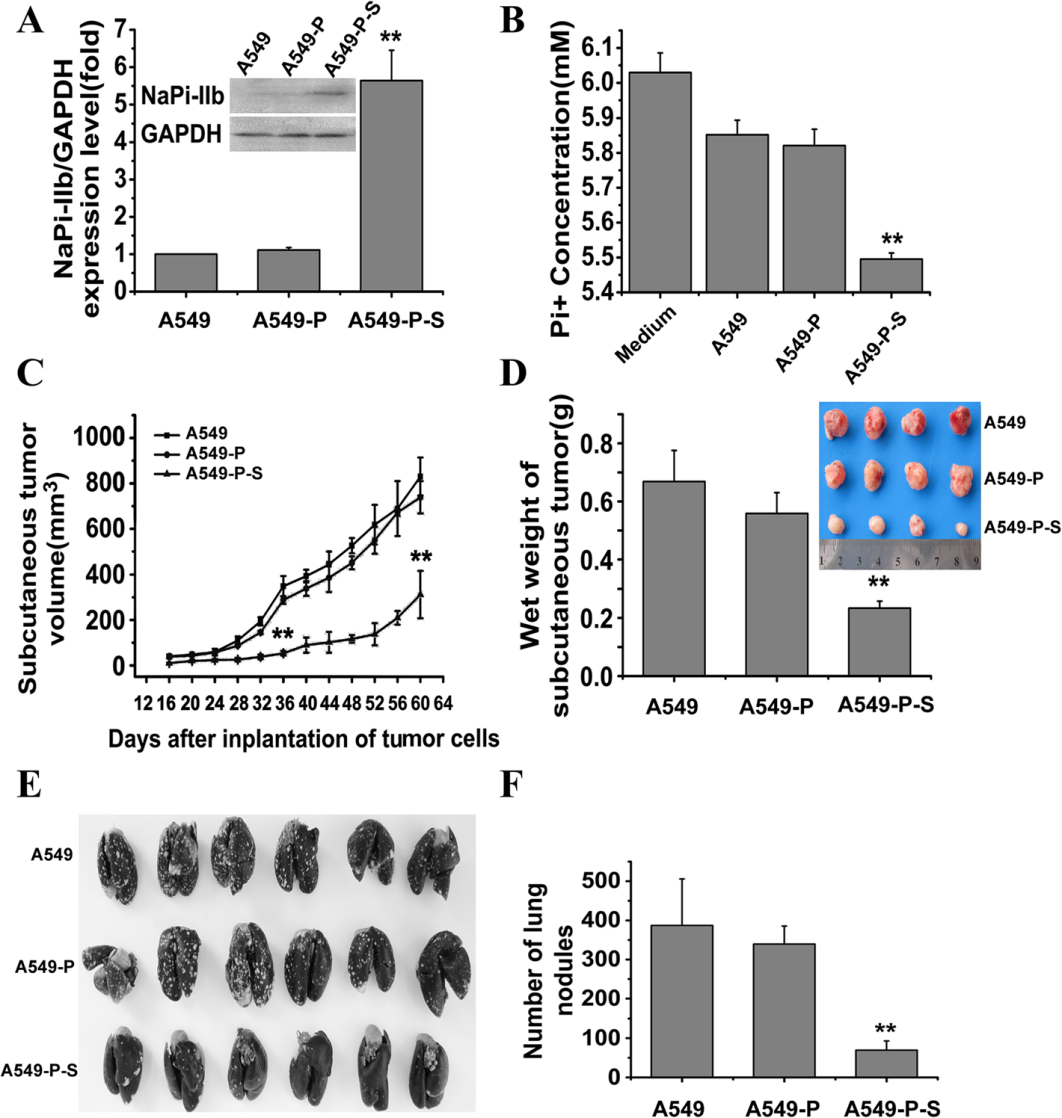
*SLC34A2* suppressed tumor growth and lung metastasis of NSCLC *in vivo*. **a** The expressions of SLC34A2 in A549-P-S, A549-P and A549 cells were confirmed by Western blot. **b** The concentrations of phosphorous in the supernatant of medium were measured by using a phosphorous measurement kit in A549-P-S, A549-P and A549 cells. **c** Tumor growth curve (each group contained 8 mice). **d** Tumor wet weight. Mice were killed for measurement of tumor weight at 60 days after inoculation. **e** Lungs from mice injected with A549-P-S, A549-P and A549 cells were injected intratracheally with India ink and fixed in AAF solution. **f** The numbers of lung nodules were counted under a dissecting microscope

In order to investigate the effects of *SLC34A2* on tumorigenesis and metastatic potential *in vivo*, A549 stable transfectants were generated by transfection with pcDNA3.1-*SLC34A2* or pcDNA3.1, and selected by G418 method. Then, western blot was done to determine the expression level of *SLC34A2* in stable cells. A549-P-S cells expressed significant high protein levels of *SLC34A2*, whereas little protein was observed in A549-P cells. The expression level of *SLC34A2* in A549-P-S cells was higher than A549 cells by 5.6-fold (Fig. 4a). To verify whether the stably transfected *SLC34A2* was functional, phosphorous concentration in the supernatant of medium was measured using a phosphorous measurement kit after stable transfection. As shown in Fig. 4b, the phosphorous level in the supernatant from the A549-P-S cells were markedly reduced compared with A549-P and A549 cells. The result confirmed the A549 cell line with stable transfection of *SLC34A2* was constructed successfully.

### Effect on *SLC34A2* on tumor growth and metastasis *in vivo*

To examine the effect of *SLC34A2* on tumor growth of NSCLC *in vivo*, a subcutaneous xenotransplanted tumor model was established with A549-P-S, A549-P or A549 cells. As shown in Fig. 4c, compared with A549 cells, A549-P-S cells exhibited a significantly slower growth rate *in vivo*. In addition, the average wet weight of tumors from nude mice injected with A549-P-S cells was less than that from nude mice injected with A549 cells (Fig. 4d). These results suggested that *SLC34A2* might play an important role in the inhibition of tumor growth *in vivo* of NSCLC.

To evaluate the effect of *SLC34A2* on tumor metastasis of NSCLC *in vivo*, a lung metastasis model in nude mice was established with A549-P-S, A549-P and A549 cells respectively. As shown in Fig. 4e, 28 days later, lungs from the mice of the three groups were injected intratrachealy with India ink and fixed in AAF solution, and the numbers of surface lung metastatic nodules were counted under a dissection microscope. We found that all of the nude mice injected with A549 or A549-P cells had shown more and larger lung nodules than nude mice injected with A549-P-S cells (Fig. 4f). These results further suggested that *SLC34A2* might play an important role in the suppression of invasion and metastasis of NSCLC.

### Effect of *SLC34A2* on expression level of related signaling proteins

**Fig. 5:**
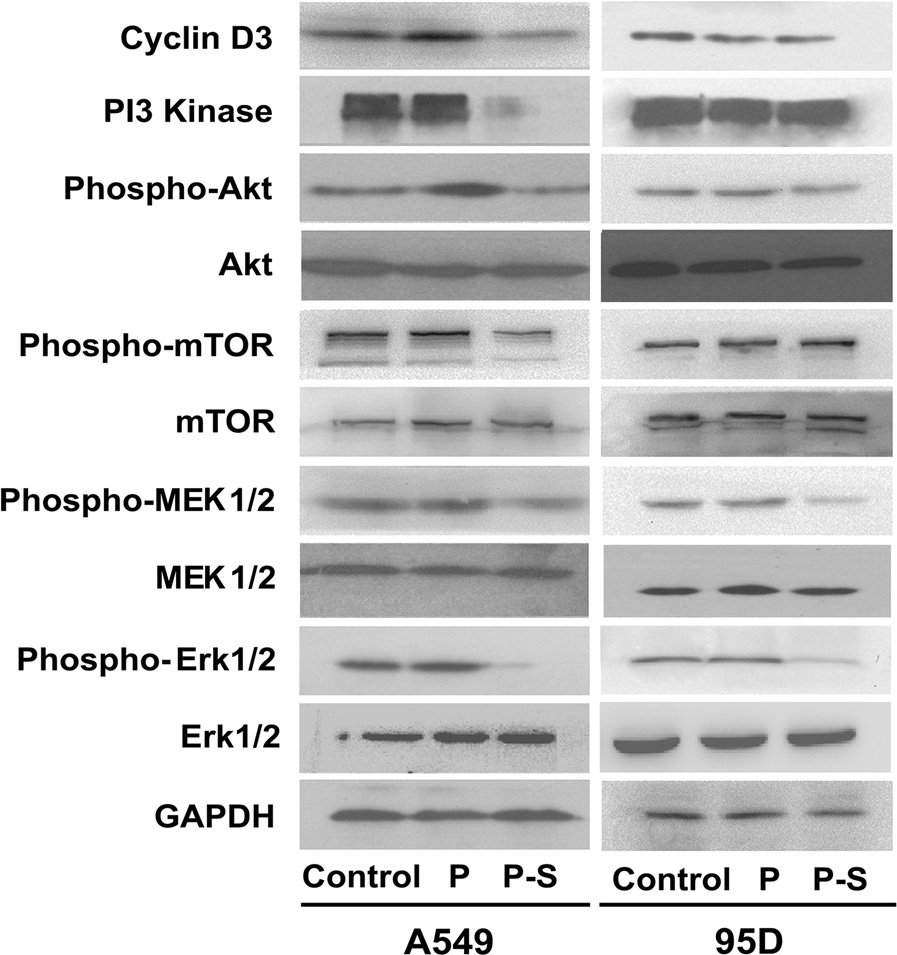
Relative signal pathways were determined by Western blot. There were not significant changes in the expressions of Akt, mTOR, MER1/2 and Erk1/2 in P-S (A549 and 95D) comparing with their control counterparts respectively. *SLC34A2*-tranfected A549 cells exhibited reduced expression levels of PI3K, p-Akt, p-mTOR, p-MEK1/2, p-Erk1/2 and CyclinD3. No significant changes in expression levels of PI3K, p-Akt, p-mTOR and CyclinD3 were found between *SLC34A2*- and vector- transfected 95D and 95D cells, whereas expressions of p-MEK1/2 and p-Erk1/2 in *SLC34A2*-transfected 95D cells were reduced (Cells transfected with pcDNA3.1-*SLC34A2* or pcDNA3.1 were named as P-S or P respectively. Untransfected cells were named as Control.)

Recent studies suggested that anomalous expression of *SLC34A2* might promote tumorigenesis of lung cancer by the activation of PI3K-Akt and Ras/Raf/MEK/ Erk pathways. To understand the mechanism of *SLC34A2* involved in tumorigenesis and progression of NSCLC, western blot was performed to detect the expression levels of related signalling protein, including PI3 Kinase, phospho-AKT, AKT phospho-mTOR, mTOR, phospho-MEK1/2, MEK1/2, phospho- Erk1/2, Erk1/2 and Cyclin D3 in A549 and 95D cells, which were transiently transfected with pcDNA3.1-*SLC34A* (P-S) or pcDNA3.1 (P) for 48 h respectively. The results showed that there were not significant changes in the expression of AKT, mTOR, MER and Erk1/2 in P-S group (A549 and 95D), comparing with their control counterparts respectively. As shown in Fig. 5, a marked reduction in PI3 Kinase, phospho-Akt, phospho-mTOR, phospho-MEK1/2, phospho- Erk1/2 and Cyclin D3 was observed in P-S cells (A549) comparing with those in control group (A549). In addition, comparing with those in control group (95D), a dramatic reduction of phospho-MEK1/2 and phospho-Erk1/2 was detected in P-S cells (95D). However, the expression levels of phospho-Akt and Cyclin D3 were slightly decreased without statistical significance. Moreover, the levels of PI3 Kinase and phospho-mTOR had no detectable changes in P-S cells (95D).

## Discussion and conclusions

In 2008, Eugene P. Kopantzev reported that the average expression level of *SLC34A2* was reduced by 9-fold compared with surrounding normal lung tissues [9]. In this report, we have also found that *SLC34A2* was down-regulated in 12 adenocarcinoma and 3 squamous carcinoma, and the lowest expression level of *SLC34A2* was decreased by 16-fold. Besides, for the first time, our results showed that *SLC34A2* was significantly down-regulated in lung adenocarcinoma cell lines (A549, H1299), bronchoalveolar carcinoma cell line (H358), lung large cell carcinoma cell lines (H460, 95D) and lung squamous carcinoma (SK-MES-1). Our results suggested *SLC34A2* with significant down-regulation in NSCLC tissues and cell lines might play an important role in tumorigenesis of NSCLC.

In our study, we further found that *SLC34A2* could perform a dramatic inhibitory effect on cell growth, motility and invasiveness in A549, H1299, 95D and SK-MES-1 cells. H358 cells are non-invasive, thus *SLC34A2* just caused growth inhibition of H358 cells. However, *SLC34A2* had no inhibition effects on cell growth, motility and invasiveness in H460 cells. Besides, *SLC34A2* did not induce any apoptosis in these six NSCLC cell lines. Moreover, we found that *SLC34A2* notably suppressed tumorigenicity and lung metastasis ability *in vivo*. For the first time, our results indicated the suppressive effects of *SLC34A2* on cell growth, motility and invasiveness in human NSCLC cell lines. It suggested that *SLC34A2* might be a new suppressor gene in NSCLC. However, *SLC34A2* might play different roles in different NSCLC cell lines.

Accumulated studies have pointed out the similarity between embryonic development and tumorigenesis [11, 12]. Genes which affect tumorigenesis usually influence embryonic development, while genes which regulate normal differentiation usually participate in tumorigenesis [13, 19]. However, the expression of these genes in tumorigenesis and progression were reported to be opposite to that in embryonic development. Genes which increased their expression levels during embryonic development usually had increased expression levels in tumorigenesis [20], such as *Cav1*. It is widely known that *Cav1* is implicated in embryonic development [21]. The low expression of *Cav1* facilitated tumorigenesis and progression of lung cancer [22]. Another example was the epidermal growth factor-Cripto-1. During early embryogenesis, Cripto-1 proteins performed an important role. Moreover, expression of Cripto-1 was increased in several human cancers and its overexpression is associated with the development of mammary tumors in mice [19]. Eugene P. Kopantzev reported the low expression level of *SLC34A2* in human NSCLC tissues. Meanwhile, our results showed that *SLC34A2* was down-regulated both in human NSCLC tissues and cell lines. Besides, Eugene P. Kopantzev also reported that *SLC34A2* was up-regulated during embryonic development. The opposite expression level of *SLC34A2* in NSCLC and embryonic development was similar to *Cav1 and* Cripto-1. It indicated that *SLC34A2* might be implicated in tumorigenesis and development of NSCLC.

To explore the pathogenesis of cancer, Reya firstly described the principles of cancer stem cell biology. It was well known that cancer stem cells might be the origin of tumor. It was derived from malignant transformation of normal stem cells after undergoing exogenous or endogenous factors [23]. Some studies showed that ATII cells were the stem cells of both ATI and ATII cells [24]. ATII cells might serve as original cells of pathogenesis of lung cancer. Some studies showed that uncontrolled proliferation of ATII cells might result in lung adenocarcinoma [25, 26]. Besides, ultrastructural and immunohistochemical studies performed in human lung cancer tissues found the presence of ATII cells [15, 16, 27–30]. TenHave-Opbroek also indicated that human ATII cells, as a pluripotential stem cell, were abnormally differentiated into ATII tumor stem cells after undergoing exogenous or endogenous factors. These tumor stem cells might invade the surrounding connective tissue and attempt to form adenocarcinoma [30]. In addition, ATII cells were also detectable in human squamous cell carcinomas and large cell carcinomas, although less frequently [16, 17, 30]. All these studies indicated that ATII cells which acted as normal stem cells, possibly transformed into cancer stem cells and ultimately induced tumorigenesis of NSCLC, especially adenocarcinoma. However, the mechanism of malignant transformation in ATII cells is unclear untill now.

For the first time, our results found that *SLC34A2* was significantly down-regulated both in NSCLC tissues and cell lines. Our further results showed that *SLC34A2* diminished cell growth, motility and invasiveness of some NSCLC cell lines *in vitro. SLC34A2* also significantly attenuated tumorigenicity and lung metastasis ability *in vivo*. These results indicated that *SLC34A2* might play an important role in the malignant transformation of ATII cells. Moreover, several studies proved a closer relationship between ATII cells and tumorigenesis of adenocarcinoma [15, 16, 27–30]. Our results also showed that *SLC34A2* was generally down-regulated in adenocarcinoma tissues and cell lines, while *SLC34A2* significantly diminished cell growth, motility and invasiveness in A549 and H1299 cells. It suggested that the dysregulation of *SLC34A2* might be more closely associated with tumorigenesis and progression of adenocarcinoma.

The role of *SLC34A2* is to transport phosphate and maintain phosphate balances [6]. Phosphate played a critical role during organ development (eg. brain, lung) and tumorigenesis through regulating cell differentiation and gene expression. Recently, Seung-Hee Chang showed that high concentration of phosphate treatment on non-tumorigenic human bronchial epithelial cells (NHBE) increased expression of sodium/phosphate co-transporter IIa (NaPi-IIa) protein and promoted cell growth through activation of Akt-mediated Raf-MEK-Erk signal pathway [31]. Besides, Hua Jin clearly demonstrated that high or low dietary phosphate could influence normal expression of *SLC34A2* and affect cell cycle and angiogenesis via activation of PI3K-Akt-mTOR signal pathway during lung development [32, 33]. Furthermore, high or low dietary phosphate increased NaPi-IIb protein levels in lungs of K-ras^LA1^ mice and stimulated lung tumorigenesis by activation of PI3K-Akt-mTOR signal pathway [34, 35]. All this studies indicated that aberrant concentration of endocellular phosphate might be relative with tumorigenesis of lung cancer. *SLC34A2* encodes the exclusive phosphate transporter protein in ATII cells, and it was vital for maintaining the balance of phosphate in ATII cells. Therefore, *SLC34A2* may be relative with tumorigenesis of lung cancer.

PI3K-Akt signal pathway which was frequently hyperactivated in lung cancer, contributed to oncogenesis and tumor growth [36]. Aberrant activation of this signal pathway promoted cell proliferation by means of modulating the function of numerous substrates, such as mammalian target of rapamycin (mTOR). Akt increased the phosphorylation of mTOR, which promoted expression of CyclinD and regulated the functions of translational regulators [37]. Several pieces of evidence attest to the promotion of hyperactivation of PI3K-Akt signal pathway in tumor cell migration and invasion [38, 39]. In addition, PI3K signaling pathway had been shown to be important to maintain pluripotency in mouse ESC. Moreover, mutations in the PI3K/AKT pathway inhibitor had been found in lung carcinomas. Activation of this pathway promoted cancer cells survival and proliferation [12]. Ras-Raf-MEK-Erk signal pathway which was also hyperactivated in lung cancer played a vital role in regulating tumor cell growth, survival and differentiation, impacting directly on the formation and progression of tumors [40]. Besides, PI3K-Akt signal pathway had been reported to cross-talk with Ras-Raf-MEK-Erk signal pathway [31]. Our results showed that expression levels of PI3 Kinase, phospho-Akt, phospho-mTOR, phospho-MEK1/2, phospho-Erk1/2 and Cyclin D3 were remarkably reduced after re-expression of *SLC34A2* in A549 cells. The results indicated that the effect of *SLC34A2* on proliferation and metastasis might depend on suppressing the activation of PI3K-Akt-mTOR and Ras-Raf-MEK-Erk signal pathways in A549 cells. However, no significant changes in expression level of PI3 Kinase, phospho-Akt, phospho-mTOR and Cyclin D3 were detected, while the levels of phospho-MEK1/2 and phospho-Erk1/2 was dramatically reduced after re-expression of *SLC34A2* in 95D cells. These results suggested that *SLC34A2* could repress the activation of Ras-Raf-MEK-Erk signal pathway, but had no effects on PI3K-Akt-mTOR signal pathway and CyclinD3 in 95D cells. Taken together, these facts indicated that the low expression of *SLC34A2* in type II alveolar epithelium cells might activate PI3K-Akt-mTOR or Ras-Raf-MEK-Erk signal pathways, and further promoted tumorigenesis of NSCLC.

In this study, for the first time, we demonstrated the general low expression of *SLC34A2* in human NSCLC tissues and cell lines. Our results supported the suppressive effects of *SLC34A2* on cell growth, motility and invasiveness in NSCLC *in vitro* and *in vivo*. We concluded that *SLC34A2* could act as a new tumor suppressor gene in NSCLC. Furthermore, our data suggested that low expression of SLC34A2 might result in tumorigenesis of NSCLC, and the relative mechanism might be related to the changes of PI3K-Akt-mTOR and Ras-Raf-MEK-Erk signal pathways. Our results will be important for understanding the early pathogenesis of human NSCLC, especially adenocarcinoma. Therefore, *SLC34A2* might provide new insights into pathogenesis of NSCLC and suggest new therapy strategy for NSCLC.

### Consent

Written informed consent was obtained from the patients for the publication of this report and any accompanying images.

## References

[1] Herbst RSHJ, Lippman SM (2008). Lung cancer. N Engl J Med.

[2] Feild JA, Zhang L, Brun KA, Brooks DP, Edwards RM (1999). Cloning and functional characterization of a sodium-dependent phosphate transporter expressed in human lung and small intestine. Biochem Biophys Res Commun.

[3] Xu H, Bai L, Collins JF, Ghishan FK (1999). Molecular cloning, functional characterization, tissue distribution, and chromosomal localization of a human, small intestinal sodium-phosphate (Na + −Pi) transporter (SLC34A2). Genomics.

[4] Yin BWT, Kiyamova R, Chua R, Caballero OL, Gout I, Gryshkova V (2008). Monoclonal antibody MX35 detects the membrane transporter NaPi2b (SLC34A2) in human carcinomas. Cancer Immun.

[5] Hashimoto M, Wang DY, Kamo T, Zhu Y, Tsujiuchi T, Konishi Y (2000). Isolation and localization of type IIb Na/Pi cotransporter in the developing rat lung. Am J Pathol.

[6] Traebert M, Hattenhauer O, Murer H, Kaissling B, Biber J (1999). Expression of type II Na-picotransporter in alveolar type II cells. Am J Physiol Lung Cell Mol Physiol.

[7] Corut A, Senyigit A, Ugur SA, Altin S, Ozcelik U, Calisir H (2006). Mutations in SLC34A2 cause pulmonary alveolar microlithiasis and are possibly associated with testicular microlithiasis. Am J Hum Genet.

[8] Cerri MF, de Rezende LCD, Paes MF, Silva IV, Rangel LBA (2010). The cotransporter NaPi-IIb: characteristics, regulation and its role in carcinogenesis. Applied Cancer Research.

[9] Kopantzev EP, Monastyrskaya GS, Vinogradova TV, Zinovyeva MV, Kostina MB, Filyukova OB (2008). Differences in gene expression levels between early and later stages of human lung development are opposite to those between normal lung tissue and non-small lung cell carcinoma. Lung Cancer.

[10] Shibasaki Y, Etoh N, Hayasaka M, Takahashi M, Kakitani M, Yamashita T (2009). Targeted deletion of the tybe IIb Na( + )−dependent Pi-co-transporter, NaPi-IIb, results in early embryonic lethality. Biochem Biophys Res Commun.

[11] Monk M, Holding C (2001). Human embryonic genes re-expressed in cancer cells. Oncogene.

[12] Dreesen O, Brivanlou AH (2007). Signaling pathways in cancer and embryonic stem cells. Stem Cell Rev Rep.

[13] Chen CM, Behringer RR (2004). Ovca1 regulates cell proliferation, embryonic development, and tumorigenesis. Genes Dev.

[14] Uhal BD (1997). Cell cycle kinetics in the alveolar epithelium. Am J Physiol Lung Cell Mol Physiol.

[15] Singh G, Katyal SL, Torikata C. Carcinoma of type II pneumocytes: immunodiagnosis of a subtype of “bronchioloalveolar carcinomas”. Am J Pathol. 1981;102:195–208. PMC1903682 6258440

[16] Kitinya JN, Sueishi K, Tanaka K, Katsuda Y. Immunoreactivity of surfactant-apoprotein in adenocarcinomas, large cell and small cell carcinomas of the lung. Acta Pathol Jpn. 1986;36:1271–8. 10.1111/j.1440-1827.1986.tb02848.x 3024446

[17] Gazdar AF, Linnoila RI, Kurita Y, Oie HK, Mulshine JL, Clark JC, et al. Peripheral airway cell differentiation in human lung cancer cell lines. Cancer Res. 1990;50:5481–7. 2386953

[18] Yang W, Wang Y, Pu Q, Ye S, Ma Q, Ren J (2014). Elevated expression of SLC34A2 inhibits the viability and invasion of A549 cells. Mol Med Rep.

[19] Strizzi L, Bianco C, Normanno N, Salomon D (2005). Cripto-1: a multifunctional modulator during embryogenesis and oncogenesis. Oncogene.

[20] Liu H, Kho AT, Kohane IS, Sun Y (2006). Predicting survival within the lung cancer histopathological hierarchy using a multi-scale genomic model of development. PLoS Med.

[21] Williams TM, Lisanti MP (2005). Caveolin-1 in oncogenic transformation, cancer, and metastasis. Am J Physiol Cell Physiol.

[22] Bélanger MM, Gaudreau M, Roussel É, Couet J (2004). Research paper role of caveolin-1 in etoposide resistance development in A549 lung cancer cells. Cancer Biol Ther.

[23] Reya T, Morrison SJ, Clarke MF, Weissman IL (2001). Stem cells, cancer, and cancer stem cells. Nature.

[24] Kinnard WV, Tuder R, Papst P, Fisher JH (1994). Regulation of alveolar type II cell differentiation and proliferation in adult rat lung explants. Am J Respir Cell Mol Biol.

[25] Thaete LG, Malkinson AM (1991). Cells of origin of primary pulmonary neoplasms in mice: morphologic and histochemical studies. Exp Lung Res.

[26] Smith B (1977). Cell line A549: a model system for the study of alveolar type II cell function. Am Rev Respir Dis.

[27] McDowell E, McLaughlin J, Merenyl D, Kieffer R, Harris C, Trump B (1978). The respiratory epithelium. V. Histogenesis of lung carcinomas in the human. J Natl Cancer Inst.

[28] Linnoila R, Mulshine J, Steinberg S, Gazdar A. Expression of surfactant-associated protein in non-small-cell lung cancer: a discriminant between biologic subsets. J Natl Cancer Inst Monogr. 1992;16:61–66. 1327036

[29] Tsutahara S, Shijubo N, Hirasawa M, Honda Y, Satoh M, Kuroki Y (1993). Lung adenocarcinoma with type II pneumocyte characteristics. Eur Respir J.

[30] Ten Have-Opbroek A, Benfield J, Van Krieken J, Dijkman J (1997). The alveolar type II cell is a pluripotential stem cell in the genesis of human adenocarcinomas and squamous cell carcinomas. Histol Histopathol.

[31] Chang SH, Yu KN, Lee YS, An GH, Beck GR, Colburn NH (2006). Elevated inorganic phosphate stimulates Akt-ERK1/2-Mnk1 signaling in human lung cells. Am J Respir Cell Mol Biol.

[32] Jin H, Chang SH, Xu CX, Shin JY, Chung YS, Park SJ (2007). High dietary inorganic phosphate affects lung through altering protein translation, cell cycle, and angiogenesis in developing mice. Toxicol Sci.

[33] Xu CX, Jin H, Chung YS, Shin JY, Hwang SK, Kwon JT, et al. Low dietary inorganic phosphate affects the lung growth of developing mice. J Vet Sci. 2009;10:105–1. 10.4142/jvs.2009.10.2.105 PMC2801121 19461205

[34] Jin H, Xu CX, Lim HT, Park SJ, Shin JY, Chung YS, et al. High dietary inorganic phosphate increases lung tumorigenesis and alters Akt signaling. Am J Respir Crit Care Med. 2009;179:59–68. 10.1164/rccm.200802-306OC PMC2615662 18849498

[35] Xu CX, Jin H, Lim HT, Ha YC, Chae CH, An GH (2010). Low dietary inorganic phosphate stimulates lung tumorigenesis through altering protein translation and cell cycle in K-ras LA1 mice. Nutr Cancer.

[36] Testa JR, Bellacosa A (2001). AKT plays a central role in tumorigenesis. Sci STKE.

[37] Hay N (2005). The Akt-mTOR tango and its relevance to cancer. Cancer Cell.

[38] Zhang D, Brodt P (2003). Type 1 insulin-like growth factor regulates MT1-MMP synthesis and tumor invasion via PI 3-kinase/Akt signaling. Oncogene.

[39] Qian Y, Corum L, Meng Q, Blenis J, Zheng JZ, Shi X (2004). PI3K induced actin filament remodeling through Akt and p70S6K1: implication of essential role in cell migration. Am J Physiol Cell Physiol.

[40] Roberts P, Der C (2007). Targeting the Raf-MEK-ERK mitogen-activated protein kinase cascade for the treatment of cancer. Oncogene.

