# Correction to: The effects and mechanisms of SLC34A2 in tumorigenesis and progression of human non-small cell lung cancer

**DOI:** 10.1186/s12929-019-0508-y

**Published:** 2019-02-13

**Authors:** Yu Wang, Weihan Yang, Qiang Pu, Yan Yang, Sujuan Ye, Qingping Ma, Jiang Ren, Zhixing Cao, Guoxing Zhong, Xuechao Zhang, Lunxu Liu, Wen Zhu

**Affiliations:** 1 State Key Laboratory of Biotherapy and Cancer Center, Collaborative Innovation Center for Biotherapy Collaborative Innovation Center for Biotherapy, West China Hospital, Sichuan University, NO. 1, Keyuan 4th Road, Gaopeng Street, High Technological Development Zone, 610041 Chengdu, Sichuan People’s Republic of China; 2 Department of Thoracic Surgery, West China Hospital, Sichuan University, No. 37 Guo Xue Xiang, 610041 Chengdu, Sichuan People’s Republic of China

**Correction to: J Biomed Sci**

**https://doi.org/10.1186/s12929-015-0158-7**

After the publication of this article [1] it came to our attention that there were some errors in two of the figures:

1. In Fig. 3, a number of images were duplicated. Specifically, in Fig. 3a we confused the SK-MES-1 control group with the P group; and in Fig. 3c we confused the A549 control group with the A549 P group. The correct images for the entire Fig. 3 have been included below. We apologise for any inconvenience this might have caused.
2. In Fig. 4 we included a Western blot image (Fig. 4a) which was identical with Fig. 1c in another article [2] by our group without making this clear in the figure legend. This has been corrected in the legend below. Again, we apologise for any inconvenienced caused.

**Fig. 3:**
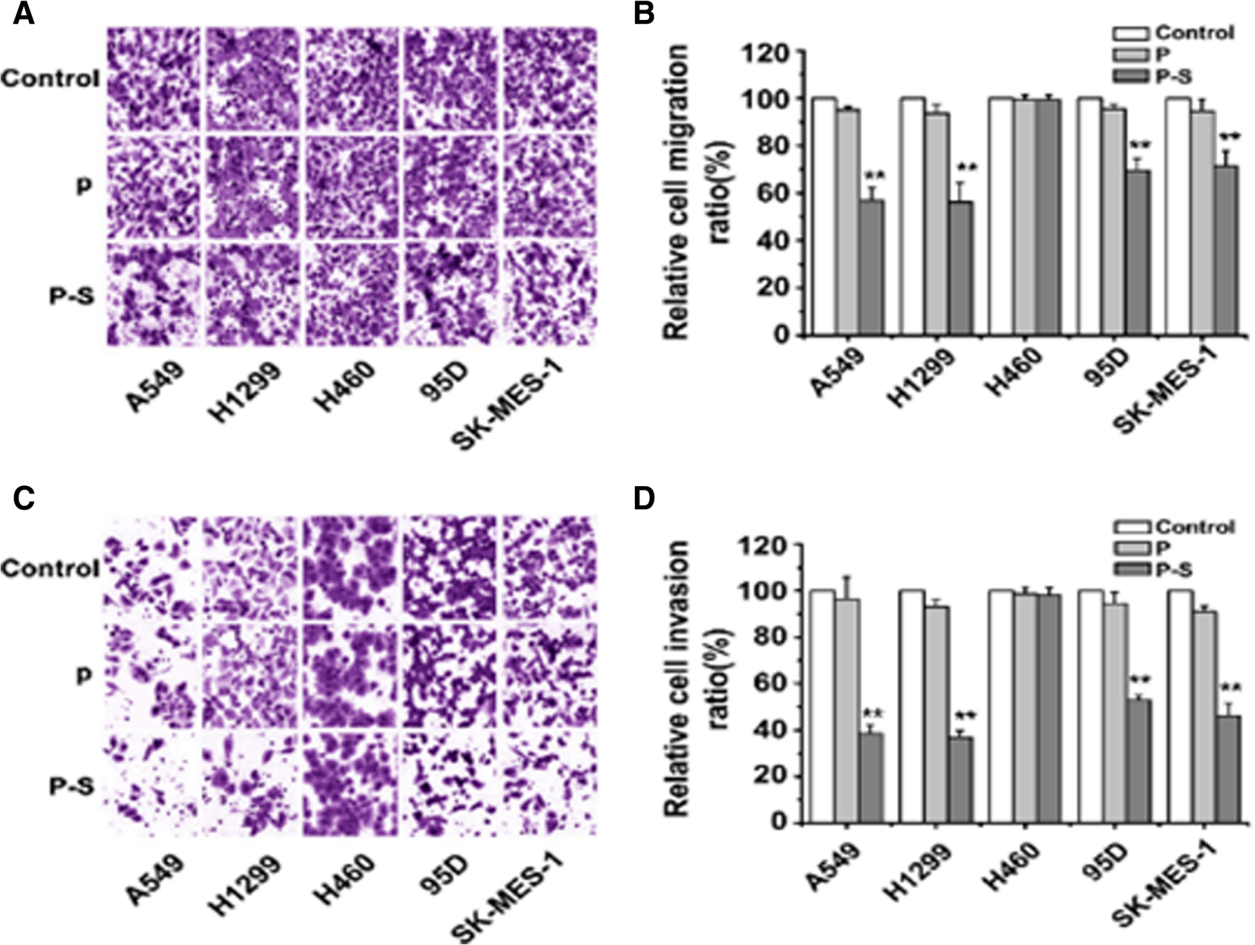
**SLC34A2 inhibited migratory and invasive potential after transient transfection for 48h.** (**a**) Millicell chamber assay showed reduced cell migratory ability in SLC34A2-transfected A549, H1299, 95D, and SK-MES-1 cells (P-S) compared with untransfected (Control) and vector- transfected cells (P). (**b**) Cell migration ratio of those five NSCLC cell lines was assessed by Matrigel invasion assay. (**c**) Matrigel invasion assay showed depressed cell invasive ability in SLC34A2-transfected A549, H1299, 95D and SK-MES-I cells compared with untransfected and vector- transfected cells. (**d**) Cell invasion ratio of those five NSCLC cell lines was assessed by Millicell chamber assay

Figure 4 SLC34A2 suppressed tumor growth and lung metastasis of NSCLC in vivo. (a) The expressions of SLC34A2 in A549-P-S, A549-P and A549 cells were confirmed by Western blot. This panel is reproduced from Yang et al. [18]. (b) The concentrations of phosphorous in the supernatant of medium were measured by using a phosphorous measurement kit in A549-P-S, A549-P and A549 cells. (c) Tumor growth curve (each group contained 8 mice). (d) Tumor wet weight. Mice were killed for measurement of tumor weight at 60 days after inoculation. (e) Lungs from mice injected with A549-P-S, A549-P and A549 cells were injected intratracheally with India ink and fixed in AAF solution. (f) The numbers of lung nodules were counted under a dissecting microscope.
